# *In situ* detection of anaerobic alkane metabolites in subsurface environments

**DOI:** 10.3389/fmicb.2013.00140

**Published:** 2013-06-04

**Authors:** Akhil Agrawal, Lisa M. Gieg

**Affiliations:** Petroleum Microbiology Research Group, Department of Biological Sciences, University of CalgaryCalgary, AB, Canada

**Keywords:** anaerobic, metabolites, alkylsuccinates, alkanes, subsurface

## Abstract

Alkanes comprise a substantial fraction of crude oil and refined fuels. As such, they are prevalent within deep subsurface fossil fuel deposits and in shallow subsurface environments such as aquifers that are contaminated with hydrocarbons. These environments are typically anaerobic, and host diverse microbial communities that can potentially use alkanes as substrates. Anaerobic alkane biodegradation has been reported to occur under nitrate-reducing, sulfate-reducing, and methanogenic conditions. Elucidating the pathways of anaerobic alkane metabolism has been of interest in order to understand how microbes can be used to remediate contaminated sites. Alkane activation primarily occurs by addition to fumarate, yielding alkylsuccinates, unique anaerobic metabolites that can be used to indicate *in situ* anaerobic alkane metabolism. These metabolites have been detected in hydrocarbon-contaminated shallow aquifers, offering strong evidence for intrinsic anaerobic bioremediation. Recently, studies have also revealed that alkylsuccinates are present in oil and coal seam production waters, indicating that anaerobic microbial communities can utilize alkanes in these deeper subsurface environments. In many crude oil reservoirs, the *in situ* anaerobic metabolism of hydrocarbons such as alkanes may be contributing to modern-day detrimental effects such as oilfield souring, or may lead to more beneficial technologies such as enhanced energy recovery from mature oilfields. In this review, we briefly describe the key metabolic pathways for anaerobic alkane (including *n*-alkanes, isoalkanes, and cyclic alkanes) metabolism and highlight several field reports wherein alkylsuccinates have provided evidence for anaerobic *in situ* alkane metabolism in shallow and deep subsurface environments.

## Introduction

Alkanes are carbon and hydrogen containing molecules that are abundant across the globe, found primarily in fossil energy deposits. They are most abundant in crude oil reservoirs, but can also be found associated with shale and coal seams (Formolo et al., 2008; Strąpoć et al., 2011). Straight chain alkanes (*n*-alkanes), branched alkanes (also known as isoalkanes), and cyclic alkanes form the saturate fraction of crude oils, which can comprise ~20% (by wt.) in a heavy oil and up to ~50% (by wt.) in a typical light oil (Hunt, 1996). The smallest alkane is methane, a C_1_ compound, while the largest alkane extends beyond C_100_ in paraffinic (waxy) oils (Hsieh et al., 2000). Under ambient conditions, alkanes ranging from C_1_–C_4_ are gases, those ranging from C_5_–C_16_ are liquids, and alkanes >C_17_ are solids (Hunt, 1996). In addition to their prevalence in petroleum mixtures, alkanes can also be found in some plants and animals, where they play a protective role or function as signaling molecules among species (Thom et al., 2007; Kunst and Samuels, 2009).

Alkanes are characterized by non-polar sigma bonds, a property that renders them relatively unreactive to most chemical transformations unless high temperatures, UV light, or specialized catalysts are applied (Carey, 2007). In contrast, these compounds are readily susceptible to microbiological transformation under ambient conditions. Understanding the biodegradation of all classes of hydrocarbons including alkanes is especially important, as the transport, storage, and usage of fossil fuel as our primary global energy source has resulted in widespread contamination of hydrocarbons into natural surface or near surface ecosystems such as groundwater aquifers. Thus, the microbial degradation of hydrocarbons represents an important fate process to mitigate such contamination. In deeper subsurface environments such as oil reservoirs, it has also been suggested that anaerobic microbial activity contributed to oil biodegradation over geological time, giving rise to heavy oil reservoirs (Huang and Larter, 2005; Head et al., 2010). Thus, understanding the processes underlying hydrocarbon biodegradation in reservoirs is important because these processes can potentially lead to detrimental effects such as heavy oil generation or reservoir souring (Voordouw et al., 2009). However, such activity can also lead to the development of beneficial biotechnologies such as microbially-enhanced energy recovery by purposefully stimulating hydrocarbon biodegradation to yield methane as an alternate energy source in mature oil reservoirs or other fossil energy environments like coal or shale deposits (Formolo et al., 2008; Gieg et al., 2008; Gray et al., 2010; Strąpoć et al., 2011). Therefore, understanding the biodegradation of hydrocarbons such as alkanes under a variety of electron-accepting conditions is important for both bioremediation and microbial-based energy recovery applications.

## Pathways of alkane biodegradation

Alkanes have long been known to be biodegradable under aerobic conditions by microorganisms. In addition to utilizing oxygen as an electron acceptor, aerobes use oxygen as a co-reactant to activate the stable carbon-carbon bonds of alkanes using monooxygenases (Rojo, 2009). The monooxygenases incorporate oxygen into the alkane molecule typically at the terminal carbon, forming an alcohol. This metabolite is then transformed to an aldehyde, which is subsequently converted to a carboxylic acid. The latter product is then readily metabolized via β-oxidation reactions to products that can enter central metabolic pathways (Rojo, 2009). The reader is referred to other papers in this volume for more details on the aerobic microbial oxidation of alkanes.

The anaerobic oxidation of alkanes has also been documented in a few early reports (ZoBell, 1946; Muller, 1957; Jack et al., 1985) but was largely discounted due to the overwhelming evidence for and widespread prevalence of aerobic alkane biodegradation processes that can readily occur in surface soils or in aerated environments. However, mounting evidence over the last two decades has shown that petroleum mixtures spilled into natural environments such as groundwater aquifers results in a rapid depletion of oxygen, creating anaerobic conditions. Thus, much attention has now also been focused on examining the potential for anaerobic hydrocarbon metabolism primarily in the interest of applying bioremediation for site cleanup.

**Figure 1:**
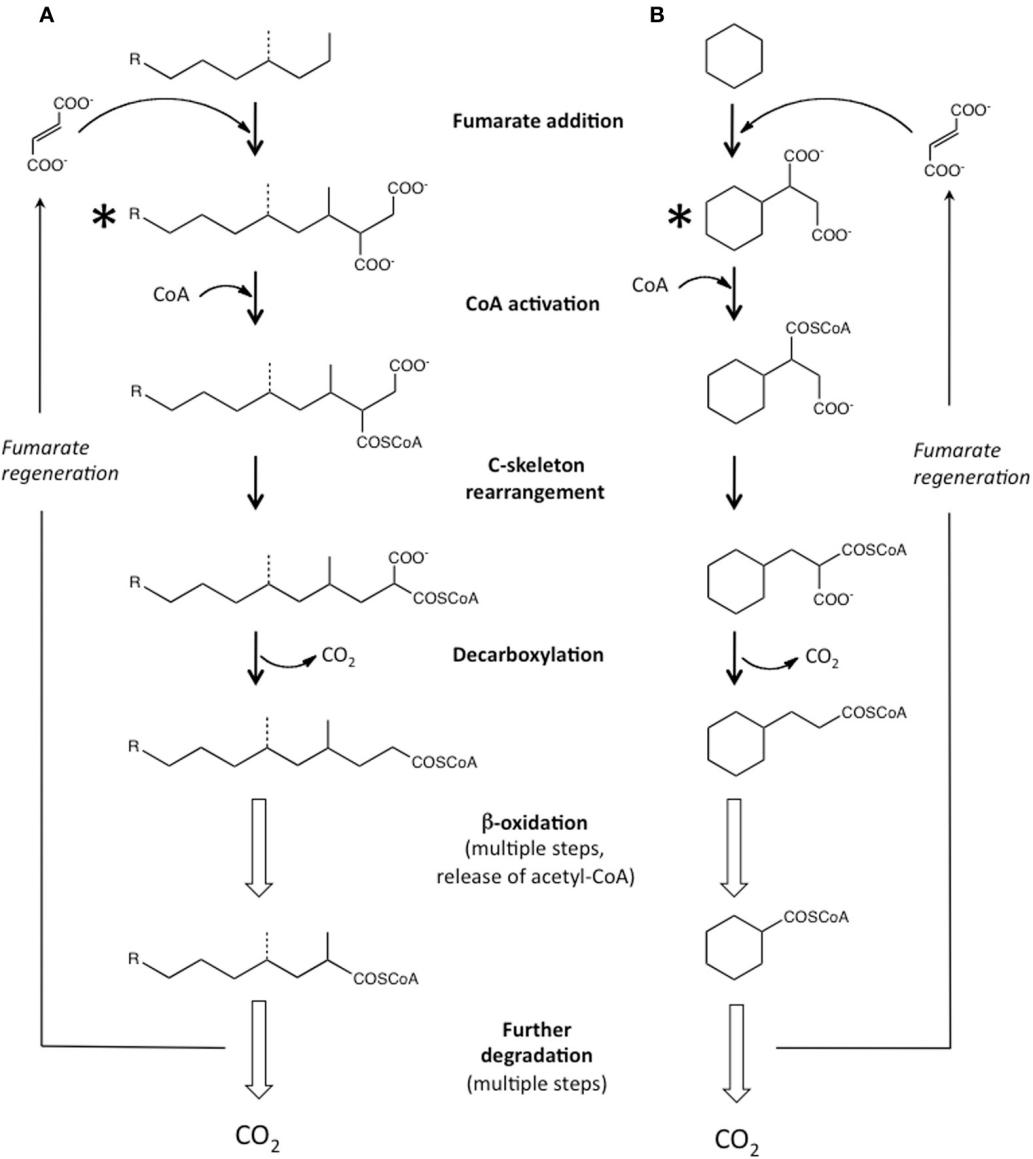
**The anaerobic biodegradation of alkanes via fumarate addition and subsequent reactions. (A)** Pathway for the biodegradation of *n*-alkanes and potentially for isoalkanes (dotted lines) based on literature reports [e.g., as overviewed by Widdel and Grundmann (2010)]. **(B)** Proposed pathway for the biodegradation of cyclic alkanes using (after Rios-Hernandez et al., 2003; Musat et al., 2010). Compounds marked with asterisks indicate fumarate addition metabolites that are most diagnostic of *in situ* anaerobic biodegradation of alkanes.

Research conducted in the 1990's examining the anaerobic biodegradation of alkylbenzenes such as toluene and xylene using samples collected from hydrocarbon-contaminated sites revealed that anaerobes activate hydrocarbons by a novel carbon-carbon addition mechanism known as “fumarate addition.” In this mechanism, the methyl group of an alkylbenzene such as toluene is added to fumarate (a C_4_-dicarboxylic acid present in central microbial metabolic pathways) forming benzylsuccinic acid (Biegert et al., 1996; Beller and Spormann, 1997). The enzyme carrying out this reaction, benzylsuccinate synthase, is known to be part of the radical SAM superfamily of enzymes (Frey, 2001; Widdel and Grundmann, 2010). Following this discovery, many more details regarding the mechanism, enzymes, and genes involved in this activation step (and further downstream metabolic reactions) have been elucidated for many aromatic hydrocarbons, including methylated mono- and polycyclic aromatic hydrocarbons [e.g., reviewed by Foght (2008)]. Subsequently, investigators examining anaerobic *n*-alkane metabolism found that this class of hydrocarbons could also be activated by carbon-carbon bond addition to fumarate, forming alkylsuccinates (Kropp et al., 2000; Rabus et al., 2001). Fumarate addition occurs primarily at the C2 subterminal carbon position of a given *n*-alkane (Figure 1A). Alkylsuccinates are then further transformed by a postulated carbon skeleton rearrangement followed by decarboxylation yielding branched fatty acids that can readily enter β-oxidation and other central metabolic pathways (Wilkes et al., 2002; Cravo-Laureau et al., 2005; Davidova et al., 2005; Callaghan et al., 2006; Widdel and Grundmann, 2010) (Figure 1A). Recently, the genes responsible for the initial “alkylsuccinate synthase” reaction were identified, known as the *masD* or *assA* genes (Callaghan et al., 2008; Grundmann et al., 2008). Laboratory-based studies examining the anaerobic degradation of pure *n*-alkanes have demonstrated that alkanes ranging from C_3_ to C_16_ are susceptible to fumarate addition, as the corresponding alkylsuccinates have been identified in culture fluids (Table 1). A recent laboratory study has also shown that *n*-alkanes up to C_26_ in whole crude oil can be degraded via fumarate addition under sulfate-reducing conditions (Aitken et al., 2013). Further, the *assA* gene has been detected in anaerobic cultures incubated with pure *n*-alkanes as short as C_3_ and as long as C_28_ (Callaghan et al., 2010). It has been suggested that fumarate addition may even be possible for anaerobic methane oxidation (Thauer and Shima, 2008; Beasley and Nanny, 2012). An alternate mechanism to fumarate addition has also been suggested involving carboxylation at the C3 position of an *n*-alkane, followed by ethyl group removal by an unknown mechanism (So et al., 2003). Studies with a nitrate-reducing enrichment culture degrading *n*-hexadecane suggested a similar mechanism (Callaghan et al., 2009). Even though recent research has shown that alkanes are biodegraded under methanogenic conditions in laboratory incubations (e.g., Zengler et al., 1999; Siddique et al., 2006; Gieg et al., 2008; Jones et al., 2008; Siddique et al., 2011; Zhou et al., 2012; Aitken et al., 2013; Cheng et al., 2013), alkylsuccinates have not been detected as metabolites during the metabolism of *n*-alkanes, prompting the hypothesis that a different pathway may be occurring under these highly reduced conditions (Mbadinga et al., 2011; Aitken et al., 2013).

**Table 1:** **Overview of literature studies wherein fumarate addition metabolites (alkylsuccinates) were detected in anaerobic laboratory incubations with *n*-alkanes added as pure substrates**.

***n*-alkane**	**Electron-accepting condition and culture**	**Literature references**
C_3_	Sulfate-reducing—strain BuS5	Kniemeyer et al., 2007
	Sulfate-reducing—enrichment culture	Savage et al., 2010
C_4_	Sulfate-reducing—strain BuS5	Kniemeyer et al., 2007
C_6_	Nitrate-reducing—strains HxN1, OcN1	Rabus et al., 2001
	Sulfate-reducing—strain ALDC (*Desulfoglaeba alkanexedens*)	Davidova et al., 2005
C_8_	Nitrate-reducing—strain HxN1	Rabus et al., 2001
	Nitrate-reducing—strains HxN1, OcN1, HdN1	Zedelius et al., 2011
	Sulfate-reducing—strain ALDC (*Desulfoglaeba alkanexedens*)	Davidova et al., 2005
C_10_	Sulfate-reducing—strain ALDC (*Desulfoglaeba alkanexedens*)	Davidova et al., 2005
	Sulfate-reducing—strain TD3	Rabus et al., 2011
C_12_	Sulfate-reducing—strain ALDC (*Desulfoglaeba alkanexedens*)	Kropp et al., 2000
		Davidova et al., 2005
C_15_	Sulfate-reducing—*Desulfatibacillum aliphaticovorans CV2803*	Cravo-Laureau et al., 2005
C_16_	Sulfate-reducing—*Desulfatibacillum aliphaticovorans CV2803*	Cravo-Laureau et al., 2005
	Sulfate-reducing—*Desulfatibacillum alkenivorans AK-01*	Callaghan et al., 2006

A few studies have now shown that cyclic alkanes may be also be activated by fumarate addition (Figure 1B). In studies examining the biotransformation of hydrocarbons in whole crude oil by a nitrate-reducer (strain HxN1) capable of utilizing C_6–C_8__ *n*-alkanes, Wilkes et al. (2003) detected metabolites showing that cyclopentane and methylcyclopentane were biotransformed to the corresponding fumarate addition products in co-metabolic reactions with hexane as the primary carbon source. Using sediments from a gas condensate-contaminated site, Rios-Hernandez et al. (2003) demonstrated sulfate-dependent ethylcyclopentane metabolism, and identified a putative fumarate addition metabolite from this cyclic alkane. In anoxic laboratory incubations prepared from the same contaminated sediments amended with sulfate and methylcyclohexane-*d*_14_, the corresponding *d*-labeled fumarate addition metabolite was also tentatively identified from this cyclic alkane (Gieg et al., 2009). Unfortunately, the exact location on the alkylated cyclic alkanes to which fumarate was added (e.g., alkyl group or ring carbon) could not be determined in the studies. Musat et al. (2010) showed that the model unsubstituted cyclic alkane cyclohexane was also activated by a carbon-carbon addition to fumarate under nitrate-reducing conditions, a process that was also coupled to anaerobic ammonium oxidation. A proposed pathway for the anaerobic biodegradation of a model cyclic alkane (cyclohexane) via fumarate addition and subsequent reactions (Rios-Hernandez et al., 2003; Musat et al., 2010) is shown in Figure 1B.

Comparatively little has been reported on the anaerobic biotransformation of isoalkanes. Branched alkanes such as pristane and phytane have frequently been used as biomarkers with which to compare the extent of biodegradation of alkanes in crude oils, as these kinds of compounds are typically more recalcitrant than *n*-alkanes (Huang and Larter, 2005). However, there is some evidence that branched alkanes can be utilized under anaerobic conditions. Two studies carried out under anaerobic conditions showed that the branched alkane pristane was susceptible to biodegradation under nitrate-reducing or methanogenic conditions but no metabolites were reported (Bregnard et al., 1997; Grossi et al., 2000). Using oil sands tailings ponds samples as an inoculum, Abu Laban et al. (2012) recently showed that C_7_ and C_8_ isoalkanes could also be metabolized under sulfate-reducing or methanogenic conditions. In the study, metabolites consistent with the mass spectral (MS) profiles of the corresponding alkylsuccinates were detected, showing for the first time that isoalkanes are also susceptible to fumarate addition (Figure 1A).

## Signature anaerobic metabolites as indicators of *in situ* hydrocarbon biodegradation

Given the overwhelming evidence that microbes are able to biodegrade hydrocarbons, relying on such activity for the remediation of contaminated sites is an attractive and relatively inexpensive clean-up option. However, documenting that such anaerobic hydrocarbon biodegradation is occurring in fuel-contaminated sites or in fossil energy reservoirs remains a challenge. For example, multiple lines of evidence, both chemical and microbiological, are required to offer support that *in situ* hydrocarbon bioremediation is occurring in contaminated aquifers (NRC, 1993; Gieg and Suflita, 2005; Beller et al., 2008; Weiss and Cozzarelli, 2008; Bombach et al., 2010; Morasch et al., 2011; Jeon and Madsen, 2012). In conjunction with the elucidation of anaerobic hydrocarbon metabolic pathways, the idea of using anaerobic metabolites to indicate that *in situ* anaerobic hydrocarbon biodegradation is occurring emerged as a powerful tool for proving *in situ* bioremediation. Beller et al. (1995) first proposed that these metabolites can be effective indicators of *in situ* anaerobic hydrocarbon biodegradation since they are uniquely anaerobic and are specific to their hydrocarbon substrate; indeed they detected these metabolites in anoxic hydrocarbon-impacted groundwater but not in uncontaminated areas. Beller (2000) proposed a list of criteria that define a signature metabolite for use as an *in situ* bioremediation indicator including that the metabolite should be (1) actively produced during biodegradation, (2) relatively chemically and biologically stable so that it can be detected, (3) a true intermediate rather than a cometabolic by-product, (4) absent in the contaminant mixture, (5) absent in uncontaminated environments, and (6) be of no commercial use.

In addition to the initial reports by Beller and colleagues, many other studies have now shown that fumarate addition metabolites of alkylbenzenes (e.g., toluene, ethylbenzene, xylenes, and trimethylbenzenes) can be detected in hydrocarbon-contaminated groundwater [see reviews by Beller (2000); Gieg and Suflita (2005), and Callaghan (2012)]. With the discovery that methylnaphthalenes and other multi-ringed aromatics (including heterocycles) can also be metabolized by fumarate addition, the corresponding succinates have also been detected in field studies (Griebler et al., 2004; Bombach et al., 2010; Jobelius et al., 2011; Morasch et al., 2011), attesting to the usefulness of this method for demonstrating the *in situ* anaerobic biodegradation of many hydrocarbon classes. Furthermore, primer sets based on the catabolic gene(s) encoding benzylsuccinate synthase have been developed by several investigators and have been used successfully to indicate the metabolic potential for microbial communities in contaminated field samples to anaerobically biodegrade alkylbenzenes (e.g., Beller et al., 2002, 2008; Winderl et al., 2007; Callaghan et al., 2010; Oka et al., 2011; von Netzer et al., 2013). It should be noted that downstream metabolites of anaerobic hydrocarbon biodegradation can also be indicators of *in situ* biodegradation, especially if they are detected in contaminated but not in unrelated, uncontaminated groundwater samples (Cozzarelli et al., 1995; Gieg and Suflita, 2002). However, these compounds are typically not specific to a parent substrate (for example, benzoate, often detected in contaminated groundwaters, is a central intermediate in the anaerobic metabolism of many aromatic compounds) or may be produced via aerobic or anaerobic processes (such as toluates from xylenes) (Beller, 2000; Gieg and Suflita, 2005). In anaerobic alkane metabolism, downstream metabolites include fatty acids, which may be produced via a variety of other cellular pathways. Thus, the fumarate addition metabolites are most diagnostic of the *in situ* anaerobic metabolism of specific hydrocarbons including alkanes. It should be noted, however, that there are some disadvantages to using signature metabolites as *sole* indicators of *in situ* bioremediation (Morasch et al., 2011). For example, these products may be below analytical detection limits, knowledge of biodegradation pathways for various hydrocarbons is required (e.g., in order to determine which metabolites to look for), and commercially-available authentic standards for many of the fumarate addition products are not available for analytical comparisons with metabolites found in field samples. Thus, using signature fumarate addition metabolites to determine whether *in situ* hydrocarbon metabolism is occurring at a given site should ideally be done in conjunction with other methods of site assessment (Bombach et al., 2010; Morasch et al., 2011).

## Anaerobic alkane metabolites in hydrocarbon-contaminated aquifers

In field bioremediation studies, the biodegradation of BTEX compounds has been a focus because these compounds are highly water-soluble (150–1780 mg/L; Heath et al., 1993) thus can rapidly migrate away from the spill source. Furthermore, benzene is among the most highly regulated hydrocarbons due to its carcinogenicity (Maltoni et al., 1989). However, in many fuel mixtures that spill into aquifers (such as gasoline, diesel, gas condensate, jet fuel), the saturate fraction (alkanes, isoalkanes, and cyclic alkanes) is the most abundant (e.g., up to 80% by wt., Heath et al., 1993). Since many alkanes have appreciable water solubilites (in the tens of mg/L), some alkanes can also migrate in groundwater away from the spill source. Many alkanes have known toxicity (Ritchie et al., 2001), thus their presence in contaminated aquifers is also of concern and their levels are often regulated (Nascarella et al., 2002).

To date, though, relatively few studies have reported on the detection of signature anaerobic alkane metabolites indicative of *in situ* anaerobic alkane in hydrocarbon-contaminated near surface environments. Gieg and Suflita (2002) documented that alkylsuccinates can be detected in hydrocarbon contaminated aquifers alongside benzylsuccinates, providing evidence that alkanes are susceptible to *in situ* anaerobic biodegradation and that alkylsuccinates can also be used as indicator metabolites. In 5 of the 6 sites examined for these signature compounds, alkylsuccinates were detected in contaminated groundwater samples, ranging from C_3_ to C_11_ alkylsuccinates depending on the site interrogated (Gieg and Suflita, 2002; Table 2). Several of the detected metabolites had MS fragments with two mass units less than those predicted to arise from straight chain (or branched) alkane metabolism, suggesting the formation of fumarate addition products from cyclic alkanes. Using sediments from a gas condensate-contaminated site, Rios-Hernandez et al. (2003) demonstrated that ethylcyclopentane could be biodegraded under sulfate-reducing conditions in laboratory incubations, and identified a putative fumarate addition metabolite that formed during the biodegradation of this compound. The gas chromatographic (GC) retention time and MS profile of this incubation-derived metabolite matched with that of a GC peak detected in several of the samples from the contaminated site, supporting the notion that cyclic alkanes can be anaerobically biotransformed *in situ*. Parisi et al. (2009) also detected alkylsuccinates from C_5_ to C_9_ *n*- or cyclic alkanes in groundwater samples collected from an aquifer contaminated with a variety of fuel mixtures while Gieg et al. (2009) detected C_5_–C_9_ alkylsuccinates in several anoxic groundwater samples prior to conducting push-pull tests in a jet fuel contaminated aquifer, further attesting to the *in situ* anaerobic biodegradation of alkanes (Table 2). In a gas condensate-contaminated site wherein alkylsuccinates were detected in groundwater samples (Gieg and Suflita, 2002; Rios-Hernandez et al., 2003), Callaghan et al. (2010) detected the presence of the alkylsuccinate synthase gene (*assA* gene), augmenting the metabolite findings. Primer sets for this gene were also used to probe samples collected from a handful of other hydrocarbon-contaminated aquifers in the US and in Germany, and indeed this gene was detected (Table 2; Callaghan et al., 2010; von Netzer et al., 2013). Recently, *assA* gene sequences were also detected in deep sea core samples collected from areas near the Deepwater Horizon oil spill, indicating that the extant organisms in deep-sea sediments have the potential to biodegrade alkanes and can potential contribute to bioremediation of such oil spills (Kimes et al., 2013). Although the alkylsuccinates themselves were not detected in these latter field sites, the detection of the gene catalyzing their formation in contaminated sites reveals a widespread potential for *in situ* anaerobic alkane biodegradation and usefulness of probing for the requisite metabolic genes. However, there are currently some limitations in the use of the *assA* gene. For example, this gene appears to be highly diverse, as a single primer set has not yet been designed that can be used to detect/amplify this gene (Callaghan et al., 2010; Aitken et al., 2013; von Netzer et al., 2013). Further, the *assA* genes show high similarity with the *bssA* genes, thus primer pairs designed to detect *assA* may also amplify the *bssA* genotypes (Callaghan et al., 2010). However, as stated above, such limitations can be overcome by coupling gene analysis with metabolite analysis and other indicators of biodegradation at field sites to provide strong evidence for intrinsic bioremediation of hydrocarbons like alkanes at contaminated sites. Table 2 summarizes the reports to date wherein alkylsuccinates (and/or alkylsuccinate synthase genes) have been detected in hydrocarbon-contaminated sites, providing evidence in support of *in situ* anaerobic alkane biodegradation.

**Table 2:** Summary of studies reporting the detection of alkylsuccinates [and/or alkylsuccinate synthase (*assA*) gene] in hydrocarbon- contaminated aquifer samples^*^.

**Field location**	**Hydrocarbon contaminant**	**Electron accepting condition(s) of field Site**	**Alkylsuccinates (and/or *assA* gene detected)**	**References**
Weld County, CO, USA	Gas condensate	Sulfate-reducing, methanogenic	Saturated: C_6_–C_9_ Unsaturated: C_6_–C_9_ *assA* gene detected	Gieg and Suflita, 2002; Rios-Hernandez et al., 2003; Callaghan et al., 2010
Wise County, TX, USA	Natural gas liquids	Sulfate-reducing	Saturated: C_3_–C_6_ Unsaturated: C_6_	Gieg and Suflita, 2002
Sedgewick County, KS, USA	Gasoline range organics	Nitrate-reducing, sulfate-reducing, methanogenic	Saturated: C_5_ Unsaturated: C_6_	Gieg and Suflita, 2002
West central AB, Canada	Flare pit site, variable hydrocarbons	Sulfate-reducing, methanogenic	Saturated: C_5_–C_11_ Unsaturated: C_7_	Gieg and Suflita, 2002
East central AB, Canada	Gas condensate	Nitrate-reducing, Fe- and Mn-reducing, sulfate-reducing	Saturated: C_4_, C_6_, C_8_ Unsaturated: C_6_, C_7_	Gieg and Suflita, 2002
Casper, WY, USA	Former refinery site, variable hydrocarbons	Sulfate-reducing, methanogenic	Saturated: C_5_, C_6_ Unsaturated: C_5_–C_9_	Parisi et al., 2009
Hickam AFB, HI, USA	Jet fuel	Sulfate-reducing, methanogenic	Saturated: C_5_–C_9_ Unsaturated: C_7_	Gieg et al., 2009
Passaic River, NJ, USA	Hydrocarbon-contaminated sediments	Not reported	*assA* gene detected	Callaghan et al., 2010
Newtown Creek, NY, USA	Hydrocarbon-contaminated sediments	Not reported	*assA* gene detected	Callaghan et al., 2010
Arthur Kill Waterway, NY/NJ, USA	Hydrocarbon-contaminated sediments	Not reported	*assA* gene detected	Callaghan et al., 2010
Gowanus Canal, NY, USA	Hydrocarbon-contaminated sediments	Not reported	*assA* gene detected	Callaghan et al., 2010
Rhine River Valley (Flingern aquifer), Germany	Former gas works site, tar oil-contaminated	Primarily sulfate-reducing	*assA* gene detected	von Netzer et al., 2013
^*^Gulf of Mexico, USA	Crude oil	Not reported, but marine sediments	*assA* gene detected	Kimes et al., 2013

* Deep-sea marine sediments rather than groundwater aquifer sediments.

## Anaerobic alkane metabolites in fossil energy reservoirs

Geochemical data suggesting the biodegradation of crude oil in reservoirs over geological time generating heavy oil has prompted an examination of these oils and fluids of oil reservoirs in general for evidence of anaerobic hydrocarbon metabolites. Upon examining 77 crude oils from across the globe, Aitken et al. (2004) detected known anaerobic metabolites of naphthalenes in 52 samples (including 2-naphthoic acid; 5,6,7,8-tetrahydro-2-naphthoic acid; decahydro-2-naphthoic acid), offering the first metabolic evidence that anaerobic *in situ* biodegradation of crude oil can occur in reservoirs. Although alkylsuccinates were also sought, none were detected in the assays. In a later study, Duncan et al. (2009) examined the metabolite profiles of several produced water samples (e.g., water removed along with oil from production wells during oil recovery) collected from high temperature Alaska North Slope (ANS) oilfields. The authors were able to detect some low molecular weight alkylsuccinates derived from C_1_–C_4_ alkanes in the produced water samples (Table 3). Downstream, branched alkanoic acids related to these alkylsuccinates were also detected (Duncan et al., 2009). Operationally, these oilfields were pressurized with natural gas to aid in oil recovery, providing the oilfield microbial community with a continuous source of low molecular weight alkanes that were likely ultimately biotransformed to the corresponding alkylsuccinates (Duncan et al., 2009). In a subsequent study, Gieg et al. (2010) examined samples collected from two different high temperature ANS oilfields, and also detected several fumarate addition metabolites including C_1_–C_3_ alkylsuccinates (Table 3). This latter study further showed that the methanogenic microbial communities present in crude oil reservoirs are able to subsist on hydrocarbons supporting the notion that oil biodegradation within fossil energy reservoirs can occur under anaerobic conditions.

**Table 3:** **Summary of studies reporting the detection of alkylsuccinates [and/or alkylsuccinate synthase (*assA*) gene] in fossil energy reservoir samples**.

**Field location**	**Field details**	**Electron accepting condition(s)**	**Alkylsuccinates (and/or *assA* gene detected)**	**References**
Alaska North Slope, USA	High temperature oilfield (50–60°C)	Sulfate-reducing, methanogenic	Saturated: C_1_–C_4_	Duncan et al., 2009
Alaska North Slope, USA	High temperature oilfield (50–60°C)	Primarily sulfate-reducing	Saturated: C_1_–C_3_	Gieg et al., 2010
Alaska North Slope, USA	High temperature oilfield (50–60°C)	Primarily methanogenic	Saturated: C_1_–C_3_	Gieg et al., 2010
SE Alberta, Canada	Low temperature oilfield (~30°C); oil produced via PWRI^1^, nitrate treatment for souring	Nitrate-reducing, sulfate-reducing, methanogenic	Saturated: C_6_–C_8_ Unsaturated: C_6_–C_8_	Agrawal et al., 2012
Huabei Oilfield, China	Low temperature oilfield (37°C)	Not reported but incubations prepared with nitrate, sulfate, and under methanogenic conditions	*assA* gene detected in methanogenic incubations from production waters	Li et al., 2012
Shengli Oilfield, China	High temperature oilfield (70°C)	Not reported but incubations prepared under methanogenic conditions	*assA* gene detected in methanogenic incubations (55°C) from production waters	Zhou et al., 2012
Shengli Oilfield, China	High temperature oilfield (70°C)	Not reported but incubations prepared under methanogenic conditions	*assA* gene detected in methanogenic hexadecane-degrading enrichment	Cheng et al., 2013
San Juan Basin (Fruitland Coal Formation, CO and NM), USA	Coal bed methane site	Methanogenic	Saturated: C_1_–C_3_, C_8_ Unsaturated: C_7_–C_8_ *ass*A gene detected	Wawrik et al., 2012

1 PWRI, Produced water re-injection.

A low temperature (~30°C) heavy oil field in Alberta, Canada (Medicine Hat Glauconitic C field) has been under study for over 5 years to test the effects of nitrate treatment on souring caused by sulfate-reducing bacteria (Lambo et al., 2008; Voordouw et al., 2009; Callbeck et al., 2011; Agrawal et al., 2012). Oil analysis conducted on various samples from this field showed that toluene served as a major electron donor driving the microbial activities within the subsurface reservoir environment (Lambo et al., 2008). This result was subsequently confirmed in laboratory studies conducted under nitrate-reducing and sulfate-reducing conditions using produced water and heavy crude oil from the field. However, the laboratory incubation tests also showed that other alkylbenzenes and alkanes present in the oil served as substrates for the oilfield microbial community (Agrawal et al., 2012). Notably, several alkanes were depleted in sulfate-reducing enrichments, and numerous metabolites indicative of fumarate addition to both aromatics and alkanes were detected, including methyl- and dimethylbenzylsuccinates and alkylsuccinates derived from C_6_–C_9_ alkanes, confirming the anaerobic biodegradation of hydrocarbons in the oil. These laboratory-based studies prompted an investigation of produced water samples from the heavy oil field for the presence of alkylsuccinates, and indeed several of these signature anaerobic alkane metabolites were detected, ranging from C_5_ to C_8_ alkylsuccinates (Table 3). These data suggested that the anaerobic microbial communities present within this low temperature reservoir can utilize alkanes as a carbon and energy source, potentially leading to souring when sulfate is present. Although alkylsuccinates were not specifically identified in other oilfields (Li et al., 2012; Zhou et al., 2012; Cheng et al., 2013), recently identified alkylsuccinate synthase genes in incubations prepared from oilfield fluids, showing that microbes residing in the sampled oilfield harbor the potential for *in situ* anaerobic alkane metabolism, again attesting to the usefulness of catabolic gene probes for determining *in situ* alkane biodegradation potential (Table 2).

Wawrik et al. (2012) recently conducted a study examining the biological conversion of coal to methane in a major coal-bed methane producing region in the USA (San Juan Basin) using a series of enrichments, functional gene assays, hydrocarbon metabolite analyses, and microbial community profiling of produced water samples. Coal is a complex, organic carbon-rich mixture derived from ancient wetland plants that were buried and transformed by heat and pressure over geological time (Strąpoć et al., 2011). While methane produced from coal beds is typically thermogenic, increasing evidence (such as the isotopic signature of methane) is showing that biologically produced methane is also occurring in many coal-bed regions (Strąpoć et al., 2011). Although not as clearly defined as crude oil, coal can contain many substrates such as alkanes (Orem et al., 2007; Strąpoć et al., 2011) that may be used by microbial communities living in coal beds to produce methane in real time, presumably via pathways similar to those shown in Figure 1. Using a functional gene array (GeoChip 3.1), Wawrik et al. (2012) detected the *assA* gene in several produced water samples collected from the San Juan Basin, showing that the indigenous microbial community has the metabolic potential for *in situ* anaerobic alkane biodegradation. In addition, they also detected C_1_–C_3_, C_7_, and C_8_ alkylsuccinates in several of the produced waters (Table 3). These data collectively showed for the first time that alkanes (and other substrates) associated with the coal can be metabolized by the associated microbial community, presumably leading to biological methane production in such fossil energy resources (Wawrik et al., 2012).

## Methods for anaerobic alkane metabolite analysis

Tools of analytical chemistry including gas chromatography-mass spectrometry (GC-MS) and high performance liquid chromatography coupled with tandem MS (LC-MS-MS) have generally been used to detect hydrocarbon metabolites in samples collected from contaminated groundwater wells or produced water wellheads of oil or coal reservoirs. Detailed descriptions of these approaches have been described in several previous publications (e.g., Beller, 2002; Gieg and Suflita, 2002, 2005; Alumbaugh et al., 2004; Jobelius et al., 2011). Briefly, for metabolite analysis by GC-MS, at least a liter of water should be collected into clean vessels (preferably glass) and acidified immediately to pH ~2 to preserve samples and protonate metabolites. Samples can then be processed in the laboratory using liquid-liquid extraction with an organic solvent such as dichloromethane, diethyl ether, or ethylacetate. Concentrated organic solvent extracts are concentrated, then derivatized by methylation or silylation prior to analysis by GC-MS (Gieg and Suflita, 2002). Depending on the method of derivatization, alkylsuccinates have diagnostic MS fragment ions that can be used to readily determine their presence in a given water sample. For example, trimethylsilyl esters of alkylsuccinates have diagnostic fragment ions of m/z 262, 217, 172, 147, and 73 (Gieg and Suflita, 2002, 2005). Metabolite concentrations can be estimated by comparison to calibration curves prepared from commercially available or readily synthesized alkylsuccinates (e.g., methylsuccinate or *n*-octylsuccinate). Detection limits for alkylsuccinates by GC-MS analysis are in the nanomolar range (Gieg and Suflita, 2002). Hydrocarbon metabolite analysis by LC-MS-MS analysis has the advantage of requiring little to no sample preparation prior to analysis thus is far less labor-intensive (Beller, 2002). Further, the technique requires only small amounts of sample (~1 mL). Parent and/or daughter ions of various metabolites are typically monitored (Beller, 2002; Alumbaugh et al., 2004). Beller (2002) found the detection limits for benzylsuccinates to be ~0.3 μg/L using LC tandem MS, while Alumbaugh et al. (2004) found that sample pre-treatment using solid-phase extraction lowered the LC-MS-MS detection level of benzylsuccinates by at least an order of magnitude (0.006–0.029 μg/L). Although high sensitivity LC-MS techniques (e.g., LC/MS-ESI-Q-TOF) have been used to successfully detect benzylsuccinates in environmental samples (Jobelius et al., 2011; Kimes et al., 2013), the detection of alkylsuccinates remains elusive, possibly because these metabolites are present at levels below detection limits (Kimes et al., 2013). Other analytical techniques such as 2-dimensional GC analysis (GC × GC) coupled with MS, currently used for separating and quantifying complex mixtures of crude oil components and related compounds (such as naphthenic acids) (e.g., Rowland et al., 2011; Reddy et al., 2012), may find future application for hydrocarbon metabolite analysis.

## Conclusions and future directions

Research on the topic of anaerobic alkane metabolism has increased in the last dozen years, revealing that alkanes can be activated by fumarate addition reactions as well as by other proposed mechanisms (yet to be understood). Of the saturate fraction of fuels, the anaerobic metabolism of *n*-alkanes has been the most frequently studied while less is known about how isoalkanes and cyclic alkanes are metabolized anaerobically, representing a knowledge gap that can be addressed by future research. As knowledge is gained regarding the key metabolic pathways for anaerobic alkane biodegradation, the identified metabolites can readily be sought in the natural environment to indicate biodegradation. Further, as newer fuel mixtures such as biofuels become more commonly used, metabolic insights gained from the study of alkanes and other hydrocarbons may help predict the kinds of metabolites that can serve as diagnostic indicators for the biotreatment of such alternative fuels. To date, a metabolite profiling approach using robust tools of analytical chemistry (GC-MS, LC-MS-MS) has been widely been used to garner evidence for the *in situ* anaerobic metabolism of aromatic hydrocarbons in contaminated aquifers, but has been used to a lesser extent for determining for *in situ* alkane biodegradation. Since alkanes (including *n*-alkanes, isoalkanes, and cyclic alkanes) comprise substantial components of many fuel mixtures that are spilled into aquifers, looking for their metabolic by-products should be included in more future site assessments to determine the prospects for *in situ* alkane bioremediation; details on how to look for such metabolites in environmental samples have been described (e.g., Gieg and Suflita, 2002; Duncan et al., 2009). Parallel assessments of the catabolic gene (e.g., *assA* gene) in subsurface microbial populations along with metabolite profiling offers very strong evidence for the *in situ* anaerobic biodegradation of alkanes (Callaghan et al., 2010; Wawrik et al., 2012), an approach that should also be part of future site assessments. Although many hydrocarbon biodegradation studies have been carried out in the context of petroleum contaminated site bioremediation, understanding anaerobic hydrocarbon biodegradation in fossil energy reservoirs has enormous economic implications related to detrimental effects (e.g., heavy oil generation and souring) and beneficial technologies (e.g., microbially enhanced energy recovery). Thus, more extensive research into hydrocarbon-related microbial metabolic processes including anaerobic alkane biodegradation in fossil energy resources such as crude oil reservoirs, and coal and shale deposits is warranted.

### Conflict of interest statement

The authors declare that the research was conducted in the absence of any commercial or financial relationships that could be construed as a potential conflict of interest.

## References

[B1] Abu Laban N. Semple K. Young R. Dao A. Foght J. M. (2012). Biodegradation of C7 and C8 iso-alkanes under methanogenic and sulfate-reducing conditions, in Abstracts of the 14th International Symposium on Microbial Ecology (Copenhagen, DK).

[B2] Agrawal A. Park H. S. Nathoo S. Gieg L. M. Jack T. R. Miner K. (2012). Toluene depletion in produced oil contributes to souring control in a fied subjected to nitrate injection. Environ. Sci. Technol. 46, 1285–1292 10.1021/es203748b 22148580

[B3] Aitken C. M. Jones D. M. Larter S. R. (2004). Anaerobic hydrocarbon biodegradation in deep subsurface oil reservoirs. Nature 341, 291–294 10.1038/nature02922 15372028

[B4] Aitken C. M. Jones D. M. Maguire M. J. Gray N. D. Sherry A. Bowler B. F. J. (2013). Evidence that crude oil alkane activation proceeds by different mechanisms under sulfate-reducing and methanogenic conditions. Geochim. Cosmochim. Acta 109, 162–174 10.1016/j.gca.2013.01.031

[B5] Alumbaugh R. E. Gieg L. M. Field J. A. (2004). Determination of alkylbenzene metabolites in groundwater by solid-phase extraction and liquid chromatography-tandem mass spectrometry. J. Chromatog. A 1042, 89–97 10.1016/j.chroma.2004.05.031 15296392

[B6] Beasley K. K. Nanny M. A. (2012). Potential energy surface for anaerobic oxidation of methane via fumarate addition. Environ. Sci. Technol. 46, 8244–8252 10.1021/es3009503 22703611

[B7] Beller H. R. (2000). Metabolic indicators for detecting *in situ* anaerobic alkylbenzene degradation. Biodegradation 11, 125–139 10.1023/A:1011109800916 11440240

[B8] Beller H. R. (2002). Analysis of benzylsuccinates in groundwater by liquid chromatography/ tandem mass spectrometry and its use for monitoring *in situ* BTEX biodegradation. Environ. Sci. Technol. 36, 2724–2728 10.1021/es025527l 12099470

[B9] Beller H. R. Ding W.-H. Reinhard M. (1995). Byproducts of anaerobic alkylbenzene metabolism useful as indicators of *in situ* bioremediation. Environ. Sci. Technol. 29, 2864–2870 10.1021/es00011a024 22206536

[B10] Beller H. R. Spormann A. (1997). Anaerobic activation of toluene and *o*-xylene by addition to fumarate in denitrifying strain T. J. Bacteriol. 179, 670–676 9006019 10.1128/jb.179.3.670-676.1997 PMC178746

[B11] Beller H. R. Kane S. R. Legler T. C. Alvarez P. J. J. (2002). A real-time polymerase chain reaction method for monitoring anaerobic, hydrocarbon-degrading bacteria based on a catabolic gene. Environ. Sci. Technol. 36, 3977–3984 10.1021/es025556w 12269751

[B12] Beller H. R. Kane S. R. Legler T. C. McKelvie J. R. Sherwood-Lollar B. Pearson F. (2008). Comparative assessments of benzene, toluene, and xylene natural attenuation by quantitative polymerase chain reaction analysis of a catabolic gene, signature metabolites, and compound-specific isotope analysis. Environ. Sci. Technol. 42, 6065–6072 10.1021/es8009666 18767667

[B13] Biegert T. Fuchs G. Heider J. (1996). Evidence that anaerobic oxidation of toluene in the denitrifying bacterium *Thauera aromatica* is initiated by formation of benzylsuccinate from toluene and fumarate. Eur. J. Biochem. 238, 661–668 10.1111/j.1432-1033.1996.0661w.x 8706665

[B14] Bombach P. Richnow H. H. Kastner M. Rischer A. (2010). Current approaches for the assessment of *in situ* biodegradation. Appl. Environ. Microbiol. 86, 839–852 10.1007/s00253-010-2461-2 20174793

[B15] Bregnard T. P. Haner A. Hohener P. Zeyer J. (1997). Anaerobic degradation of pristane in nitrate-reducing microcosms and enrichment cultures. Appl. Environ. Microbiol. 63, 2077–2081 16535616 10.1128/aem.63.5.2077-2081.1997 PMC1389171

[B16] Callaghan A. V. (2012). Metabolomic investigations of anaerobic hydrocarbon-impacted environments. Curr. Opin. Biotechnol. [Epub ahead of print]. 10.1016/j.copbio.2012.08.012 22999828

[B17] Callaghan A. V. Davidova I. A. Savage-Ashlock K. Parisi V. A. Gieg L. M. Suflita J. M. (2010). Diversity of benzyl- and alkylsuccinate synthase genes in hydrocarbon-impacted environments and enrichment cultures. Environ. Sci. Technol. 44, 7287–7294 10.1021/es1002023 20504044

[B18] Callaghan A. V. Gieg L. M. Kropp K. G. Suflita J. M. Young L. Y. (2006). Comparison of mechanisms of alkane metabolism under sulfate-reducing conditions among two bacterial isolates and a bacterial consortium. Appl. Environ. Microbiol. 72, 4274–4282 10.1128/AEM.02896-05 16751542 PMC1489600

[B19] Callaghan A. V. Tierney M. Phelps C. D. Young L. Y. (2009). Anaerobic biodegradation of n-hexadecane by a nitrate-reducing consortium. Appl. Environ. Microbiol. 75, 1339–1344 10.1128/AEM.02491-08 19114507 PMC2648152

[B20] Callaghan A. V. Wawrik B. Ní Chadhain S. M. Young L. Y. Zylstra G. J. (2008). Anaerobic alkane-degrading strain AK-01 contains two alkylsuccinate synthase genes. Biochem. Biophys. Res. Commun. 366, 142–148 10.1016/j.bbrc.2007.11.094 18053803

[B21] Callbeck C. M. Dong X. Chatterjee I. Agrawal A. Caffrey S. M. Sensen C. (2011). Microbial community succession in a bioreactor modeling a souring low temperature oil reservoir subjected to nitrate injection. Appl. Microbiol. Biotech. 91, 799–810 10.1007/s00253-011-3287-2 21538114

[B22] Carey F. A. (2007). Organic Chemistry, 7th Edn. New York, NY: McGraw-Hill Science, 1312.

[B23] Cheng L. Rui J. Li Q. Zhang H. Lu Y. (2013). Enrichment and dynamics of novel syntrophs in a methanogenic hexadecane-degrading culture from a Chinese oilfield. FEMS Microbiol. Ecol. 83, 757–766 10.1111/1574-6941.12031 23066709

[B24] Cozzarelli I. M. Herman J. S. Baedecker M. J. (1995). Fate of microbial metabolites of hydrocarbons in a coastal plain aquifer: the role of electron acceptors. Environ. Sci. Technol. 29, 458–469 10.1021/es00002a023 22201393

[B25] Cravo-Laureau C. Grossi V. Raphel D. Matheron R. Hirschler-Rea A. (2005). Anaerobic *n*-alkane metabolism by a sulfate-reducing bacterium, *Desulfatibacillum aliphaticovorans* strain CV2803^T^. Appl. Environ. Microbiol. 71, 3458–3467 10.1128/AEM.71.7.3458-3467.2005 16000749 PMC1169040

[B26] Davidova I. A. Gieg L. M. Nanny M. Kropp K. G. Suflita J. M. (2005). Stable isotopic studies of *n*-alkane metabolism by a sulfate-reducing bacterial enrichment culture. Appl. Environ. Microbiol. 71, 8174–8182 10.1128/AEM.71.12.8174-8182.2005 16332800 PMC1317434

[B27] Duncan K. E. Gieg L. M. Parisi V. A. Tanner R. S. Tringe S. G. Bristow J. (2009). Biocorrosive thermophilic microbial communities in Alaskan North Slope oil facilities. Environ. Sci. Technol. 43, 7977–7984 10.1021/es9013932 19921923

[B28] Foght J. (2008). Anaerobic biodegradation of aromatic hydrocarbons: pathways and prospects. J. Mol. Microbiol. Biotechnol. 15, 93–120 10.1159/000121324 18685265

[B29] Formolo M. J. Salacup J. M. Petsch S. T. Martini A. M. Nusslein K. (2008). A new model linking atmospheric methane sources to Pleistocene glaciation via methanogenesis in sedimentary basins. Geology 36, 139–142

[B30] Frey P. A. (2001). Radical mechanisms of enzymatic catalysis. Ann. Rev. Biochem. 70, 121–148 10.1146/annurev.biochem.70.1.121 11395404

[B31] Gieg L. M. Alumbaugh R. E. Field J. A. Jones J. Istok J. D. Suflita J. M. (2009). Assessing *in situ* rates of anaerobic hydrocarbon bioremediation. Microb. Biotechnol. 2, 222–233 10.1111/j.1751-7915.2008.00081.x 21261916 PMC3815842

[B32] Gieg L. M. Davidova I. A. Duncan K. E. Suflita J. M. (2010). Methanogenesis, sulfate reduction and crude oil biodegradation in hot Alaskan oilfields. Environ. Microbiol. 12, 3074–3086 10.1111/j.1462-2920.2010.02282.x 20602630

[B33] Gieg L. M. Duncan K. E. Suflita J. M. (2008). Bioenergy production via microbial conversion of residual oil to natural gas. Appl. Environ. Microbiol. 74, 3022–3029 10.1128/AEM.00119-08 18378655 PMC2394919

[B34] Gieg L. M. Suflita J. M. (2002). Detection of anaerobic metabolites of saturated and aromatic hydrocarbons in petroleum-contaminated aquifers. Environ. Sci. Technol. 36, 3755–3762 12322748 10.1021/es0205333

[B35] Gieg L. M. Suflita J. M. (2005). Metabolic indicators of anaerobic hydrocarbon biodegradation in petroleum-laden environments, in Petroleum Microbiology. eds Ollivier B. Magot M. (Washington DC: ASM Press), 337–356

[B36] Gray N. D. Sherry A. Hubert C. Dolfing J. Head I. M. (2010). Methanogenic degradation of petroleum hydrocarbons in subsurface environments: remediation, heavy oil formation, and energy recovery. Adv. Appl. Microbiol. 72, 137–161 10.1016/S0065-2164(10)72005-0 20602990

[B37] Griebler C. Safinowski M. Vieth A. Richnow H. H. Meckenstock R. U. (2004). Combined stable carbon isotope analysis and specific metabolites determination for assessing is situ degradation of aromatic hydrocarbons in a tar oil-contaminated aquifer. Environ. Sci. Technol. 38, 617–631 14750740 10.1021/es0344516

[B38] Grossi V. Raphel D. Hirschler-Rea A. Gilewicz M. Mouzdahir A. Bertrand J.-C. (2000). Anaerobic biodegradation of pristane by a marine sedimentary bacterial and/or archaeal community. Org. Geochem. 31, 769–772 10.1016/S0146-6380(00)00060-7

[B39] Grundmann O. Behrends A. Rabus R. Amann J. Halder T. Heider J. (2008). Genes encoding the candidate enzyme encoding for anaerobic activation of *n*-alkanes in the denitrifying bacterium, strain HxN1. Environ. Microbiol. 10, 376–385 10.1111/j.1462-2920.2007.01458.x 17961174

[B40] Head I. M. Larter S. R. Gray N. D. Sherry A. Adams J. J. Aitken C. M. (2010). Hydrocarbon biodegradation in petroleum reservoirs, in Handbook of Hydrocarbon and Lipid Microbiology, ed Timmis T. M. (Berlin: Springer-Verlag), 3098–3109

[B41] Heath J. S. Koblis K. Sager S. L. (1993). Review of chemical, physical, and toxicologic properties of components of total petroleum hydrocarbons. J. Soil Contam. 2, 1–25

[B42] Hsieh M. Philp R. P. del Rio J. C. (2000). Characterization of high molecular weight biomarkers in crude oils. Org. Geochem. 31, 1581–1588

[B43] Huang H. Larter S. R. (2005). Biodegradation of petroleum in subsurface geological reservoirs, in Petroleum Microbiology, eds Ollivier B. Magot M. (Washington, DC: ASM Press), 91–121

[B44] Hunt J. M. (1996). Petroleum Geochemistry and Geology, 2nd Edn. New York, NY: W. H. Freeman and Company

[B45] Jack T. R. Lee E. Mueller J. (1985). Anaerobic gas production from crude oil, in Microbes and Oil Recovery: International Bioresources Journal, Vol. 1, eds Zajic J. E. Donaldson E. C. (El Paso, TX: Bioresources Publications), 167–180

[B46] Jeon C. O. Madsen E. L. (2012). *in situ* microbial metabolism of aromatic-hydrocarbon environmental pollutants. Curr. Opin. Biotechnol. Epub ahead of print]. 10.1016/j.copbio.2012.09.001 22999827

[B47] Jobelius C. Ruth B. Griebler C. Meckenstock R. U. Hollender J. Reineke A. (2011). Metabolites indicate hot spots of biodegradation and biogeochemical gradients in a high-resolution monitoring well. Environ. Sci. Technol. 45, 474–481 10.1021/es1030867 21121661

[B48] Jones D. M. Head I. A. Gray N. D. Adams J. J. Rowan A. K. Aitken C. M. (2008). Crude oil biodegradation via methanogenesis in subsurface petroleum reservoirs. Nature 451, 176–180 10.1038/nature06484 18075503

[B49] Kimes N. E. Callaghan A. V. Aktas D. F. Smith W. L. Sunner J. Golding B. T. (2013). Metagenomic analysis and metabolite profiling of deep-sea sediments from the Gulf of Mexico following the Deepwater Horizon oil spill. Front. Microbiol. 4:50 10.3389/fmicb.2013.00050 23508965 PMC3598227

[B50] Kniemeyer O. Musat F. Sievert S. M. Knittel K. Wilkes H. Blumenberg M. (2007). Anaerobic oxidation of short-chain hydrocarbons by marine sulphate-reducing bacteria. Nature 449, 898–902 10.1038/nature06200 17882164

[B51] Kropp K. G. Davidova I. A. Suflita J. M. (2000). Anaerobic oxidation of *n*-dodecane by an addition reaction in a sulfate-reducing bacterial enrichment culture. Appl. Environ. Microbiol. 66, 5393–5398 10.1128/AEM.66.12.5393-5398.2000 11097919 PMC92473

[B52] Kunst L. Samuels L. (2009). Plant cuticles shine: advances in wax biosynthesis and export. Curr. Opin. Plant Biol. 12, 721–727 10.1016/j.pbi.2009.09.009 19864175

[B53] Lambo A. J. Noke K. Larter S. R. Voordouw G. (2008). Competitive, microbially-mediated reduction of nitrate with sulfide and aromatic oil components in a low-temperature, western Canadian oil reservoir. Environ. Sci. Technol. 42, 8941–8946 19192822 10.1021/es801832s

[B54] Li W. Wang L.-Y. Duan R.-Y. Liu J.-F. Gu J.-D. Mu B.-Z. (2012). Microbial community characteristics of petroleum reservoir production water amended with *n*-alkanes and incubated under nitrate-, sulfate-reducing and methanogenic conditions. Int. Biodeter. Biodeg. 69, 87–96 10.1016/j.ibiod.2012.01.005

[B55] Maltoni C. Ciliberti A. Cotti G. Conti B. Belpoggi F. (1989). Benzene, an experimental multipotential carcinogen: results of the long-term bioassays performed at the Bologna Institute of Oncology. Environ. Health Perspect. 82, 109–124 2792037 10.1289/ehp.8982109 PMC1568122

[B56] Mbadinga S. M. Wang L.-Y. Zhou L. Liu J.-F. Gu J.-D. Mu B.-Z. (2011). Microbial communities involved in anaerobic degradation of alkanes. Int. Biodeter. Biodeg. 65, 1–13 10.1016/j.ibiod.2010.11.009

[B57] Morasch B. Hunkeler D. Zopfi J. Temime B. Hohener P. (2011). Intrinsic biodegradation potential of aromatic hydrocarbons in an alluvial aquifer—potentials and limits of signature metabolite analysis and two stable isotope-based techniques. Water Res. 45, 4459–4469 10.1016/j.watres.2011.05.040 21741669

[B58] Muller F. M. (1957). On methane fermentation of higher alkanes. Antonie Van Leeuwenhoek 23, 369–384 10.1007/BF02545890 13509644

[B59] Musat F. Wilkes H. Behrends A. Woebken D. Widdel F. (2010). Microbial nitrate-dependent cyclohexane degradation coupled with anaerobic ammonium oxidation. ISME J. 4, 1290–1301 10.1038/ismej.2010.50 20410937

[B60] Nascarella M. A. Kostecki P. T. Calabrese E. J. Click D. (2002). AEHS's 2001 survey of states' soil and groundwater cleanup standards, in Contaminated Soil, Sediment, and Water (January/February), 15–68

[B61] National Research Council (NRC). (1993). In Situ Bioremediation: When Does it Work? Washington, DC: National Academy Press

[B62] Oka A. R. Phelps C. D. Zhu X. Saber D. L. Young L. Y. (2011). Dual biomarkers of anaerobic hydrocarbon degradation in historically contaminated groundwater. Environ. Sci. Technol. 45, 3407–3414 10.1021/es103859t 21438602

[B63] Orem W. H. Tatu C. A. Lerch H. E. Rice C. A. Bartos T. T. Bates A. L. (2007). Organic compounds in produced waters from coalbed natural gas wells in the Powder River Basin, Wyoming, USA. Appl. Geochem. 22, 2240–2256 19452885

[B64] Parisi V. A. Brubaker G. R. Zenker M. J. Prince R. C. Gieg L. M. da Silva M. L. B. (2009). Field metabolomics and laboratory assessments of anaerobic intrinsic bioremediation of hydrocarbons at a petroleum-contaminated site. Microb. Biotechnol. 2, 202–212 10.1111/j.1751-7915.2009.00077.x 21261914 PMC3815840

[B65] Rabus R. Jarling R. Lahme S. Kuhner S. Heider J. Widdel F. (2011). Co-metabolic conversion of toluene in anaerobic *n*-alkane-degrading bacteria. Environ. Microbiol. 13, 2576–2586 10.1111/j.1462-2920.2011.02529.x 21880102

[B66] Rabus R. Wilkes H. Behrends A. Armstroff A. Fischer T. Pierik A. J. (2001). Anaerobic initial reaction of *n*-alkanes in a denitrifying bacterium: evidence for (1-methylpentyl)succinate as initial product and for involvement of an organic radical in *n*-hexane metabolism. J. Bacteriol. 183, 1707–1715 10.1128/JB.183.5.1707-1715.2001 11160102 PMC95056

[B67] Reddy C. M. Arey J. S. Seewald J. S. Sylva S. Lemkau K. L. Nelson R. K. (2012). Composition and fate of gas and oil released to the water column during the Deepwater Horizon oil spill. Proc. Natl. Acad. Sci. U.S.A. 109, 20229–20234 10.1073/pnas.1101242108 21768331 PMC3528605

[B68] Rios-Hernandez L. A. Gieg L. M. Suflita J. M. (2003). Biodegradation of an alicyclic hydrocarbon by a sulfate-reducing enrichment from a gas condensate-contaminated aquifer. Appl. Environ. Microbiol. 69, 434–443 10.1128/AEM.69.1.434-443.2003 12514025 PMC152447

[B69] Ritchie G. D. Still K. R. Alexander W. K. Nordholm A. F. Wilson C. L. Rossi J. 3rd. (2001). A review of the neurotoxicity risk of selected hydrocarbon fuels. J. Toxicol. Environ. Health 4(Pt. B), 223–312 10.1080/10937400118874 11503417

[B70] Rojo F. (2009). Degradation of alkanes by bacteria. Environ. Microbiol. 11, 2477–2490 10.1111/j.1462-2920.2009.01948.x 19807712

[B71] Rowland S. J. West C. E. Scarlett A. G. Jones D. Frank R. A. (2011). Identification of individual tetra− and pentacyclic naphthenic acids in oil sands process water by comprehensive two−dimensional gas chromatography/mass spectrometry. Rapid Commun. Mass Spectrom. 25, 1198–1204 10.1002/rcm.4977 21488118

[B72] Savage K. N. Krumholz L. R. Gieg L. M. Parisi V. A. Suflita J. M. Allen J. (2010). Biodegradation of low-molecular-weight alkanes under mesophilic sulfate-reducing conditions: metabolic intermediates and community patterns. FEMS Microbiol. Ecol. 72, 485–495 10.1111/j.1574-6941.2010.00866.x 20402777

[B73] Siddique T. Fedorak P. M. Foght J. M. (2006). Biodegradation of short-chain *n*-alkanes in oil sands tailings ponds under methanogenic conditions. Environ. Sci. Technol. 40, 5459–5464 10.1021/es200649t 16999125

[B74] Siddique T. Penner T. Semple K. Foght J. M. (2011). Anaerobic biodegradation of longer chain *n*-alkanes coupled to methane production in oil sands tailings. Environ. Sci. Technol. 45, 5892–5899 10.1021/es200649t 21644510

[B75] So C. M. Phelps C. D. Young L. Y. (2003). Anaerobic transformation of alkanes to fatty acids by a sulfate-reducing bacterium, strain Hxd3. Appl. Environ. Microbiol. 69, 3892–3900 10.1128/AEM.69.7.3892-3900.2003 12839758 PMC165127

[B76] Strąpoć D. Mastalerz M. Dawson K. Macaladay J. L. Callaghan A. V. Wawrik B. (2011). Biogeochemistry of microbial coal-bed methane. Annu. Rev. Earth Planet. Sci. 39, 617–656

[B77] Thauer R. K. Shima S. (2008). Methane as fuel for anaerobic microorganisms. Ann. N.Y. Acad. Sci. 1125, 158–170 10.1196/annals.1419.000 18096853

[B78] Thom C. Gilley D. C. Hooper J. Esch H. E. (2007). The scent of the waggle dance. PLoS Biol. 5:e228 10.1371/journal.pbio.0050228 17713987 PMC1994260

[B79] von Netzer F. Pilloni G. Kleindienst S. Krueger M. Knittel K. Grundger T. (2013). Enhanced gene detection assays for fumarate-adding enzymes allow uncovering anaerobic hydrocarbon degraders in terrestrial and marine systems. Appl. Environ. Microbiol. 79, 543–552 10.1128/AEM.02362-12 23124238 PMC3553772

[B80] Voordouw G. Grigoryan A. A. Lambo A. Lin S. Park H. S. Jack T. R. (2009). Sulfide remediation by pulsed injection of nitrate into a low temperature Canadian heavy oil reservoir. Environ. Sci. Technol. 43, 9512–9518 10.1021/es902211j 20000549

[B81] Wawrik B. Mendivelso M. Parisi V. A. Suflita J. M. Davidova I. A. Marks C. R. (2012). Field and laboratory studies on the bioconversion of coal to methane in the San Juan Basin. FEMS Microbiol. Ecol. 81, 26–42 10.1111/j.1574-6941.2011.01272.x 22146015

[B82] Weiss J. V. Cozzarelli I. M. (2008). Biodegradation in contaminated aquifers: incorporating microbial/molecular methods. Ground Water 46, 305–322 10.1111/j.1745-6584.2007.00409.x 18194318

[B83] Widdel F. Grundmann O. (2010). Biochemistry of the anaerobic degradation of non-methane alkanes, in Handbook of Hydrocarbon and Lipid Microbiology, ed Timmis K. N. (Berlin: Springer-Verlag), 909–924

[B84] Wilkes H. Kuhner S. Bolm C. Fischer T. Classen A. Widdel F. (2003). Formation of *n*-alkane- and cycloalkane-derived organic acids during anaerobic growth of a denitrifying bacterium with crude oil. Org. Geochem. 34, 1313–1323

[B85] Wilkes H. Rabus R. Fischer T. Armstroff A. Behrends A. Widdel F. (2002). Anaerobic degradation of *n*-hexane in a denitrifying bacterium: further degradation of the initial intermediate (1-methylpentyl)succinate via C-skeleton rearrangement. Arch. Microbiol. 177, 235–243 10.1007/s00203-001-0381-3 11907679

[B86] Winderl C. Schaefer S. Lueders T. (2007). Detection of anaerobic toluene and hydrocarbon degraders in contaminated aquifers using benzylsuccinate synthase (bssA) genes as a functional marker. Environ. Microbiol. 9, 1035–1046 10.1111/j.1462-2920.2006.01230.x 17359274

[B87] Zedelius J. Rabus R. Grundmann O. Werner I. Brodkorb D. Schreiber F. (2011). Alkane degradation under anoxic conditions by a nitrate-reducing bacterium with possible involvement of the electron acceptor in substrate activation. Environ. Microbiol. Rep. 3, 125–135 10.1111/j.1758-2229.2010.00198.x 21837252 PMC3151549

[B88] Zengler K. Richnow H. H. Rossello-Mora R. Michaelis W. Widdel F. (1999). Methane formation from long-chain alkanes by anaerobic microorganisms. Nature 401, 266–269 10.1038/45777 10499582

[B89] Zhou L. Li K.-P. Mbadinga S. M. Yang S.-Z. Gu J.-D. Mu B.-Z. (2012). Analyses of *n*-alkanes degrading community dynamics of a high-temperature methanogenic consortium enriched from production water of a petroleum reservoir by a combination of molecular techniques. Exotoxicology 21, 1680–1691 10.1007/s10646-012-0949-5 22688358

[B90] ZoBell C. E. (1946). Action of microorganisms on hydrocarbons. Bacteriol. Rev. 10, 1–49 20996704

